# The longitudinal relationship between subjective exercise experience and physical exercise among university students: the moderating role of motivational beliefs

**DOI:** 10.3389/fpubh.2026.1972061

**Published:** 2026-10-29

**Authors:** Delu Meng, Wenlong Guo, Ting Zhang, Xinqi He, Peng Jia, Jing Li, Luzhen Ding

**Affiliations:** 1 School of Physical Education, Jiangxi Normal University, Nanchang, Jiangxi, China; 2 Admissions and Career Services, Jiangxi Institute of Construction, Nanchang, Jiangxi, China

**Keywords:** cross-lagged analysis, motivational beliefs, physical exercise behavior, subjective exercise experience, university students

## Abstract

**Purpose:**

To investigate the longitudinal relationship between university students’ subjective exercise experiences and their physical exercise behavior using a cross-lagged research design, and to analyze the moderating role of motivational beliefs in this relationship.

**Methods:**

A one-year, two-stage longitudinal questionnaire survey was conducted among 422 university students in Jiangxi Province (52.6% male, mean age = 18.29 ± 0.632 years) using the Subjective Exercise Experience Scale, the Adolescent Physical Activity Scale, the Exercise Self-Efficacy Scale and the Perceived Value of Exercise Scale.

**Results:**

(1) Gender differences were observed in both subjective exercise experience and physical exercise behavior across the two phases; male participants scored higher on average than female participants across all measures. (2) There was a significant positive correlation between subjective exercise experience and physical exercise behavior among university students. (3) Results from a cross-lagged model controlling for gender indicated that subjective exercise experience at T1 significantly and positively predicted physical exercise behavior at T2 (*β* = 0.385, *p* < 0.001), and physical exercise behavior at T1 significantly and positively predicted subjective exercise experience at T2 (*β* = 0.336, *p* < 0.001). (4) Motivational beliefs exerted a significant positive moderating effect on both cross-lagged pathways.

**Conclusion:**

Gender differences exist in both subjective exercise experience and physical exercise behavior among university students; there is a longitudinal relationship across time between subjective exercise experience and physical exercise behavior; and motivational beliefs exert a significant moderating effect on the prospective predictive pathways between subjective exercise experience and physical exercise behavior.

## 1 Introduction

As the most dynamic and creative group of young people, the physical and mental health of university students is not only crucial to their personal growth and development, but is also closely linked to the quality of the nation’s talent development and the future of the nation (1–3). However, insufficient physical activity remains a significant public health issue worldwide, with approximately one quarter of adults globally failing to meet the recommended standards for physical activity (4). The physical health of university students in China also faces challenges, with issues such as insufficient participation in physical exercise and difficulties in establishing exercise habits being relatively common (5). Policy documents such as the ‘Healthy China 2030’ Outline and the ‘Opinions on Comprehensively Strengthening and Improving School Physical Education in the New Era’ explicitly state that physical education must be incorporated into the core curriculum for talent development at the higher education level, and that targeted physical activity interventions should be implemented to address prominent health issues among young people (6, 7). Traditional strategies to promote physical exercise among university students have largely focused on external institutional interventions, such as compulsory physical education courses and quantified exercise targets. While such measures can increase exercise frequency in the short term, they often fail to translate into long-term, stable and self-directed behavioral habits due to a lack of intrinsic psychological motivation (8). A meta-analysis of interventions by Ntoumanis et al. (9) further demonstrates that external control alone is insufficient to sustain healthy behaviors; autonomous motivation and intrinsic experience are the key determinants of behavioral persistence. Consequently, an increasing number of researchers have begun to focus on subjective psychological experiences during exercise, suggesting that pleasure, a sense of achievement and vitality may serve as important internal reinforcers driving individuals to participate repeatedly (10–14).

Subjective exercise experience refers to the immediate, emotional and subjective evaluations and feelings an individual has regarding their own physiological and psychological state during and after physical exercise (15, 16). From the perspective of behavioral drive mechanisms, hedonistic theory posits that when making decisions, individuals anticipate the emotional consequences of a behavior and tend to choose options that maximize positive emotions and minimize negative ones (17). The affect-reflection theory further suggests that existing emotional experiences reshape the cognitive memory system and influence subsequent behavioral decisions; positive experiences elicit approach behavior, while negative experiences trigger avoidance behavior, and a bidirectional dynamic relationship exists between stimuli and responses (18). Cross-sectional studies have confirmed that subjective exercise experiences are significantly positively correlated with physical exercise behavior; positive experiences help to promote exercise behavior, while sustained exercise also helps to enhance positive experiences (10–13). Exercise intervention studies have also found that enhancing the enjoyment of exercise helps to promote regular exercise (14). Longitudinal evidence is also available: Williams et al. (19) found that acute emotional responses to moderate-intensity exercise could predict physical activity participation at 6 and 12 months. A systematic review by Rhodes and Kates also indicates that emotional responses during exercise can predict future exercise motivation and behavior (20). With regard to cross-lagged studies, Xu and Dong (21) employed cross-lagged analysis and found that adolescents’ subjective exercise experiences and leisure exercise habits exhibited a stable positive correlation across time, and that the two variables had a reciprocal predictive relationship. Dong et al. (22) also found, in a sample of adolescents, that subjective exercise experience was associated with exercise behavior across time, and that exercise behavior could predict subsequent subjective exercise experience; Duan et al. (23), using a sample of university students, found that subjective exercise experience played a significant mediating role between the exercise environment and exercise behavior. These studies have preliminarily revealed the longitudinal association between subjective exercise experience and exercise behavior; however, significant gaps remain: existing cross-lagged panel studies have largely focused on antecedent variables such as adolescents’ leisure-time exercise, peer relationships, and exercise climate, while the bidirectional relationship between ‘experience → behavior’ and ‘behavior → experience’ over a one-year interval among university students has yet to be adequately examined; More importantly, these studies have not simultaneously tested the moderating role of motivational beliefs in the two cross-lagged pathways, nor have they examined the moderating effects of exercise self-efficacy and perceived value of exercise on these pathways separately. Therefore, the novelty of this study lies not in the repeated use of a longitudinal design, but in incorporating motivational beliefs into a cross-lagged panel model to examine their conditional moderation of the longitudinal relationship between subjective exercise experience and physical exercise behavior.

Cross-lagged panel models, by controlling for the autoregressive effects of outcome variables from the previous time period while simultaneously estimating the two cross-lagged paths “experience → behavior” and “behavior → experience”—are suitable for testing bidirectional cross-temporal relationships among variables and have been applied in the field of exercise behavior (21). However, traditional cross-lagged panel models may confound between-individual differences with within-individual changes; based on this, Hamaker et al. (24) proposed the random-intercept cross-lagged model. After comparing various cross-lagged models, Orth et al. (25) also noted that different models yield varying inferences regarding the direction of causality; Mund and Nestler (26) further emphasized that the appropriate model should be selected based on the research question and data waves. This study has only two waves of data collected 1 year apart, which is insufficient to reliably estimate a random-intercept cross-lagged panel model; therefore, we employ a traditional cross-lagged panel model for preliminary testing and exercise caution when interpreting the direction of causality. More importantly, this study focuses not only on the “longitudinal design” itself but also on whether motivational beliefs can alter the strength of the cross-temporal transition between experience and behavior. Motivational beliefs are systematic cognitive judgments individuals make regarding their own abilities and the value of a task in achievement contexts; they are core psychological antecedents that drive behavioral choices, effort investment, and behavioral persistence (27). According to the expectancy-value theory, motivational beliefs can be divided into expectancy beliefs and task-value beliefs: the former manifests in the context of exercise as exercise self-efficacy—that is, an individual’s confidence in overcoming obstacles such as time, physical fitness, and the environment to persist in exercise; the latter corresponds to the perception of the value of exercise, that is, an individual’s recognition of and emphasis on the value of exercise in promoting health and regulating emotions (28–30). The tripartite interaction theory of social cognition posits that an individual’s cognition, behavior, and environment mutually shape one another; cognitive factors not only influence behavior but also affect an individual’s processing and interpretation of emotional experiences (31). Previous research has found that exercise self-efficacy can serve as a moderator between exercise intention and behavior (32), and individuals with high self-efficacy are better at using strategies such as cognitive reappraisal to manage emotional experiences (33, 34). Perceptions of the value of exercise, meanwhile, may prompt individuals to interpret exercise experiences as valuable emotional experiences, thereby sustaining positive experiences and participation (35). This evidence suggests that motivational beliefs may play a moderating role in the longitudinal relationship between subjective exercise experiences and physical exercise behavior. Specifically, motivational beliefs may primarily influence the reciprocal transformation process between experience and behavior. Regarding the stability of these two variables over time, on the one hand, individuals with high motivational beliefs may be better at maintaining established patterns; on the other hand, habit theory suggests that once an action becomes automated, its maintenance relies more on situational cues than on motivational evaluations (36, 37). The exact nature of this moderating effect remains unclear and requires further testing.

Based on the aforementioned theoretical and empirical research, this study employs a two-phase cross-lagged design with a one-year interval to examine the longitudinal relationship between university students’ subjective exercise experience and physical exercise behavior, and to test the moderating effects of exercise self-efficacy and perceived value of exercise, respectively. The following hypotheses are proposed (Figure 1): H1: There are concurrent and sequential correlations between university students’ subjective exercise experience and physical exercise behavior at T1 and T2; H2: Subjective exercise experience at T1 predicts physical exercise behavior at T2; H3: Physical exercise behavior at T1 predicts subjective exercise experience at T2; H4a: Exercise self-efficacy moderates the path from subjective exercise experience at T1 to subjective exercise experience at T2; H4b: Perceived value of exercise moderates the path from subjective exercise experience at T1 to subjective exercise experience at T2; H5a: Exercise self-efficacy moderates the path from T1 physical exercise behavior to T2 physical exercise behavior; H5b: Perceived value of exercise moderates the path from T1 physical exercise behavior to T2 physical exercise behavior; H6a: Exercise self-efficacy moderates the path from T1 subjective exercise experience to T2 physical exercise behavior; H6b: Perceived value of exercise moderates the path from T1 subjective exercise experience to T2 physical exercise behavior; H7a: Exercise self-efficacy moderates the path from T1 physical exercise behavior to T2 subjective exercise experience; H7b: Perceived value of exercise moderates the path from T1 physical exercise behavior to T2 subjective exercise experience.

**Figure 1:**
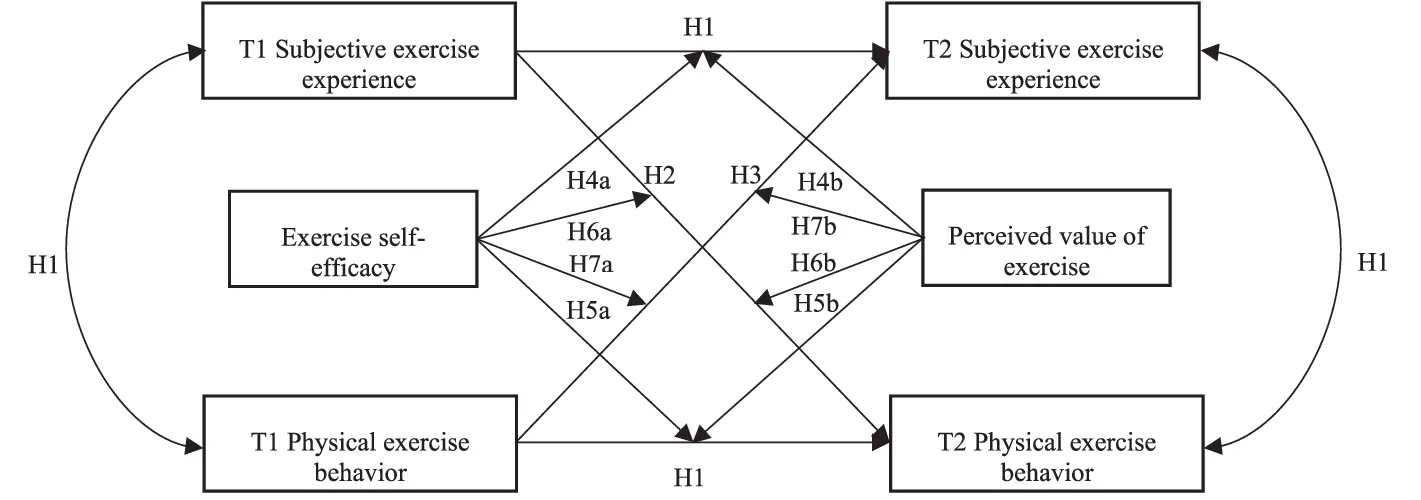
Hypothesis model. H4a–H7b correspond to the moderating effect hypotheses of exercise self-efficacy and perceived value of exercise on the four pathways, respectively.

## 2 Materials and methods

### 2.1 Objects of study

This study employed convenience sampling, selecting six universities in Nanchang and Jiujiang, Jiangxi Province, as the study sites. A two-phase longitudinal survey, conducted 1 year apart, was administered to first-year university students in 18 classes who were not majoring in physical education. All questionnaires were administered using paper-and-pencil tests. To facilitate the matching of data from the two surveys, each participant was assigned a unique code. The sample tracking and matching process is shown in Figure 2. At T1, 518 questionnaires were collected; after excluding 22 invalid questionnaires, 496 valid questionnaires remained. At T2, 460 questionnaires were collected; after excluding 20 invalid questionnaires, 440 valid questionnaires remained. The valid T1 and T2 questionnaires were matched using unique codes. Among them, 64 T1 questionnaires and 8 T2 questionnaires could not be matched, resulting in 432 successfully matched questionnaires. During the second round of screening and review, a further 10 invalid questionnaires were excluded, yielding a final valid sample of 422 participants (overall response rate: 81.47%), including 222 males (52.6%) and 200 females (47.4%). The mean age of the final valid sample was 18.29 ± 0.632 years. Analysis and testing revealed no significant differences between the analysis sample and the dropout sample in terms of gender (*χ^2^* = 0.076, *p* = 0.782), T1 physical exercise behavior (*t* = 1.380, *p* = 0.168), T1 subjective exercise experience (*t* = −0.701, *p* = 0.484), exercise self-efficacy (*t* = −1.102, *p* = 0.271), and perceived value of exercise (*t* = 0.733, *p* = 0.464), indicating that the attrition of participants between the two surveys was non-structural. Given that the core model of this study was a cross-lagged panel model, Monte Carlo simulations (Mplus 8.3, 1,000 replications) were conducted as a *post hoc*, data-dependent power assessment for the main cross-lagged paths at *N* = 422 (38, 39). The population parameters in the simulations were specified using unstandardized coefficients estimated from the observed dataset (40). Assuming that the observed effects represented the true population effects, the estimated power for all main cross-lagged paths was 1.000, exceeding the conventional threshold of 0.80 (41). Given that students were nested within classes, this study calculated the ICC(1) with classes as the clustering unit. The results showed that the ICC(1) for each variable ranged from 0.001 to 0.003, with design effects ranging from 1.02 to 1.07. According to the criteria established by Kreft and De Leeuw (42), this study exhibits a small clustering effect. Furthermore, all design effects were less than 2, indicating that the clustering effect at the class level had a limited impact on the inflation of standard errors, and that analyses at the individual level were not subject to significant bias. Consequently, no cluster correction was applied to the subsequent cross-lagged and moderation models (43). This study has been approved by an ethics review board, and no personal information, such as participants’ names, was recorded during the questionnaire collection process.

**Figure 2:**
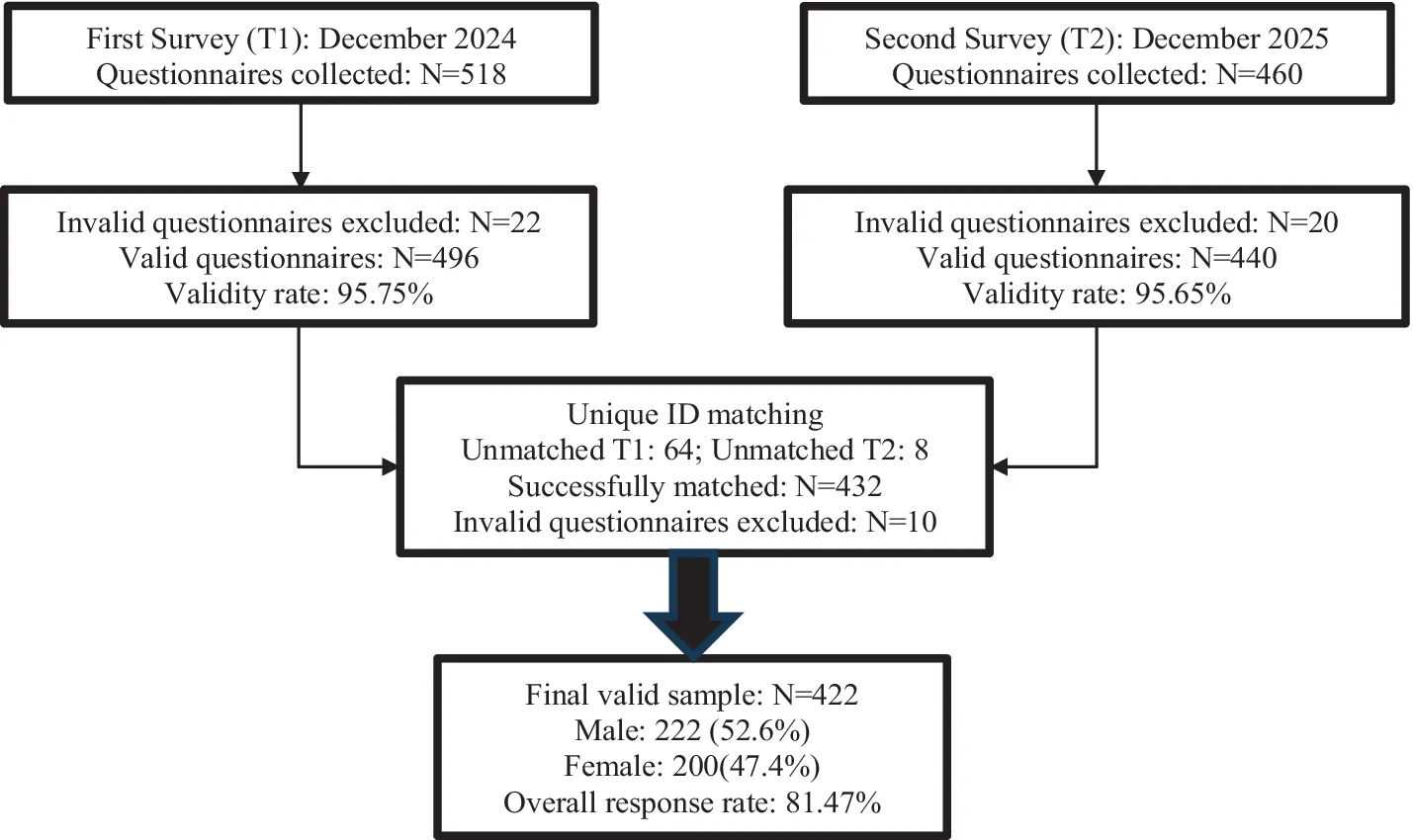
Flowchart of participant recruitment and retention in the study. Invalid questionnaires were excluded based on the following criteria: more than one-third of items were missing; answers were obviously consistent; or responses followed a pattern. The second round of review was also based on the above criteria. Reasons for non-matching included school transfers and absences.

### 2.2 Measuring tools

#### 2.2.1 Subjective exercise experiences scale

The “Positive Well-being” and “Psychological Distress” sections of the Subjective Exercise Experiences Scale developed by McAuley and Courneya (44) were used. Each section contained 4 items, for a total of 8 items. A 5-point Likert scale was used (1 = “Strongly Disagree” to 5 = “Strongly Agree”). Positive well-being was positively scored, and psychological distress was reverse-scored; scores ranged from 8 to 40, with higher scores indicating a better subjective exercise experience. In this study, the two dimensions of positive well-being and psychological distress were highly correlated, and because the present study focused on overall subjective exercise experience, a second-order measurement model was adopted. In this model, the eight items loaded onto two first-order factors, which in turn loaded onto one second-order factor representing overall subjective exercise experience. This second-order model was used consistently in the longitudinal measurement invariance tests, the cross-lagged model, and the latent moderation model (10). In this study, the Cronbach’s alpha coefficients for this scale were 0.917 (T1) and 0.928 (T2); according to De Vellis’s recommendations, the internal consistency is good. The valid sample size for this scale in this study was 422. The absolute values of item skewness for the two surveys were T1: 0.296–0.563 and T2: 0.206–0.357; the absolute values of item kurtosis were T1: 0.178–0.653 and T2: 0.045–0.563; the minimum standard deviation was 0.936 (T1) and 0.903 (T2). The fit indices from confirmatory factor analysis were good (T1: *χ^2^/df* = 1.581, CFI = 0.997, TLI = 0.994, RMSEA = 0.037, SRMR = 0.016; T2: *χ^2^/df* = 1.924, CFI = 0.996, TLI = 0.991, RMSEA = 0.047, SRMR = 0.012). This scale is suitable for the sample under study.

#### 2.2.2 Physical exercise behavior scale

The “Physical Activity Rating Scale for Adolescents” (PARS-3), revised by Liang Deqing, was used to assess the participants’ physical exercise behavior (45), which evaluates exercise volume based on three aspects: duration, frequency, and intensity. All three dimensions use a 5-point Likert scale; higher scores indicate greater exercise volume. The total exercise score is calculated as intensity × (duration – 1) × frequency, with a score range of 0–100; a higher score indicates a greater total exercise volume and higher levels of physical exercise behavior. This scale has been validated by scholars as having good criterion-related validity (*p* = 0.82) (45). In this study, the valid sample size for this scale was 422. The absolute values of skewness for each item across the two surveys were T1: 0.105–0.411 and T2: 0.045–0.952; the absolute values of kurtosis were T1: 0.970–1.358 and T2: 0.411–1.542; the minimum standard deviations were 1.153 (T1) and 0.962 (T2). Since the PARS-3 total score is calculated using the formula “intensity × (time − 1) × frequency”—a formula that combines three dimensions, each of which reflects different aspects of physical exercise rather than a single homogeneous construct—traditional confirmatory factor analysis (CFA) is not applicable; therefore, no CFA was conducted. This scale has been validated by numerous studies as having good reliability and validity, and its composite total score is widely used in the field of sports science to assess exercise behavior (46).

#### 2.2.3 Exercise self-efficacy scale

This study adopted the “Exercise Self-Efficacy Scale” (47) developed by Wu, Ronis, and Pender and revised by Wang et al. (48). To align with the exercise context of university students, the term “sports” in some items of the original scale was replaced with “physical exercise.” This scale has been validated among university students by Dong et al. (49). The scale consists of 12 items, is unidimensional, and uses a 3-point Likert scale ranging from 1 (“I cannot do it”) to 3 (“I am certain I can do it”). The score range is 12–36, with higher scores indicating higher exercise self-efficacy. In this study, exercise self-efficacy was measured at T1 only, the valid sample size for this scale was 422, and Cronbach’s alpha was 0.902. The absolute values of skewness for each item ranged from 0.113 to 0.974; the absolute values of kurtosis ranged from 0.088 to 1.150; the minimum standard deviation was 0.618. The fit indices from confirmatory factor analysis were good (*χ^2^/df* = 2.787, CFI = 0.960, TLI = 0.946, RMSEA = 0.065, SRMR = 0.036). This scale is suitable for the sample under study.

#### 2.2.4 Perceived value of exercise scale

The “Perceived Value of Exercise Scale” developed by Kirk (50) was adopted. After translation, back-translation, and expert review, the scale was revised for use with a university student sample; Jia et al. (51) have previously validated its construct validity among Chinese university students. The scale consists of 7 items scored using a 5-point Likert scale (1 = “Disagree” to 5 = “Agree”). The score range is 7–35, with higher scores indicating a higher level of perceived value of exercise. In this study, perceived value of exercise was measured at T1 only, the valid sample size for this scale was 422, and Cronbach’s alpha was 0.887. The absolute values of skewness for each item ranged from 0.041 to 0.645; the absolute values of kurtosis ranged from 0.068 to 0.928; and the minimum standard deviation was 0.785. The fit indices for confirmatory factor analysis were good (*χ^2^/df* = 2.928, CFI = 0.989, TLI = 0.976, RMSEA = 0.068, SRMR = 0.024). The scale is suitable for the sample under study.

The results for reliability, skewness, kurtosis, and CFA fit indices for each scale are summarized in Table 1. Cronbach’s alpha for all scales was greater than 0.88; the maximum absolute value of skewness was 0.950, and the maximum absolute value of kurtosis was 1.542; all CFA fit indices met the standards recommended by Hu and Bentler (52).

**Table 1:** Summary of reliability, skewness, kurtosis, and CFA fit indices for each scale.

Variable	Cronbach’s alpha	Absolute skewness	Absolute kurtosis	*χ^2^/df*	CFI	TLI	RMSEA	SRMR
T1 Subjective exercise experience	0.917	0.296 ~ 0.563	0.178 ~ 0.653	1.581	0.997	0.994	0.037	0.016
T2 Subjective exercise experience	0.928	0.206 ~ 0.357	0.045 ~ 0.563	1.924	0.996	0.991	0.047	0.012
T1 Physical exercise behavior	–	0.105 ~ 0.411	0.970 ~ 1.358	–	–	–	–	–
T2 Physical exercise behavior	–	0.045 ~ 0.952	0.411 ~ 1.542	–	–	–	–	–
Exercise self-efficacy	0.902	0.113 ~ 0.974	0.088 ~ 1.150	2.787	0.960	0.946	0.065	0.036
Perceived value of exercise	0.887	0.041 ~ 0.645	0.068 ~ 0.928	2.928	0.989	0.976	0.068	0.024

Cronbach’s alpha criteria:α ≥ 0.80 is considered good, and α ≥ 0.90 is considered excellent (67). Skewness and kurtosis criteria: absolute value of skewness < 3, absolute value of kurtosis < 10 (68). CFA fit criteria: CFI/TLI ≥ 0.90, RMSEA ≤ 0.08, SRMR ≤ 0.08 (52), and *χ*^2^/df < 3 (68).

### 2.3 Data analysis

SPSS 27 and Mplus 8.3 software were used to perform descriptive statistics, reliability analysis, normality tests, confirmatory factor analysis, common method bias tests, correlation analysis, measurement invariance tests, difference analysis, and longitudinal structural equation modeling.

## 3 Results

### 3.1 Common method bias test

This study endeavored to minimize common-method bias through both procedural controls and statistical tests. Procedurally, both surveys employed anonymous paper-and-pencil administration, used unique codes for matching, provided standardized instructions, were administered 1 year apart, and included reverse-scored items on some scales. Statistically, a Harman one-factor test was first conducted; the first factor in the unrotated exploratory factor analysis explained 27.892% of the variance, which is below the commonly used reference standard of 40%. Further confirmatory factor analysis was conducted by assigning all measurement indicators to a single common-method factor; the model fit was poor: *χ^2^/df* = 8.55, CFI = 0.382, TLI = 0.349, RMSEA = 0.134, and SRMR = 0.133. It should be noted that Harman’s single-factor test has low sensitivity; the poor model fit of the single common-method factor model merely indicates that the data are not dominated by a single common-method factor, but does not prove the absence of common-method bias. Therefore, this study maintains caution regarding the interpretation of correlations among variables, particularly concurrent correlations. Since the core of this study is to control for cross-lagged paths after accounting for autoregression, and given the one-year interval between the two phases, common-method bias is unlikely to fully account for the cross-temporal predictive relationships; however, its influence cannot be completely ruled out (53).

### 3.2 Correlation analysis

The results of the correlation analysis (Table 2) show that there were significant positive correlations between subjective exercise experience at T1 and T2 (*r* = 0.483) and between physical exercise behavior at T1 and T2 (*r* = 0.572). Furthermore, there were significant positive correlations between subjective exercise experience at T1 and physical exercise behavior at T1 (*r* = 0.425), between subjective exercise experience at T2 and physical exercise behavior at T2 (*r* = 0.507), T1 subjective exercise experience and T2 physical exercise behavior (*r* = 0.510), and T1 physical exercise behavior and T2 subjective exercise experience (*r* = 0.520) were all significantly positively correlated. Exercise self-efficacy was significantly positively correlated with T1 subjective exercise experience (*r* = 0.266), subjective exercise experience at T2 (*r* = 0.233), physical exercise behavior at T1 (*r* = 0.292) and physical exercise behavior at T2 (*r* = 0.349) were all significantly positively correlated; perceived value of exercise was significantly positively correlated with subjective exercise experience at T1 (*r* = 0.399), subjective exercise experience at T2 (*r* = 0.393), T1 physical exercise behavior (*r* = 0.369) and T2 physical exercise behavior (*r* = 0.428); exercise self-efficacy was significantly positively correlated with perceived value of exercise (*r* = 0.361). This indicates that university students’ physical exercise behavior and subjective exercise experience exhibit concurrent correlations and stability across time, while motivational beliefs are significantly positively correlated with physical exercise behavior and subjective exercise experience at all stages.

**Table 2:** Means, standard deviations, and intercorrelations for the main study variables.

Variable	Mean	SD	1	2	3	4	5	6
1. T1 Subjective exercise experience	28.65	6.33	–					
2. T2 Subjective exercise experience	28.07	6.19	0.483***	–				
3. T1 Physical exercise behavior	21.71	19.41	0.425***	0.520***	–			
4. T2 Physical exercise behavior	22.39	18.72	0.510***	0.507***	0.572***	–		
5. Exercise self-efficacy	20.86	5.84	0.266***	0.233***	0.292***	0.349***	–	
6. Perceived value of exercise	24.31	4.43	0.399***	0.393***	0.369***	0.428***	0.361***	–

*p < 0.05, **p < 0.01, ***p < 0.001.

### 3.3 Longitudinal measurement invariance analysis

We conducted tests of longitudinal measurement invariance (configural, metric, scalar, and strict invariance) for subjective exercise experience. The longitudinal measurement invariance tests were based on the second-order measurement model of subjective exercise experience. The results indicate that (Table 3) subjective exercise experience satisfies all four levels of invariance—configural, metric, scalar, and strict (ΔCFI ≤ 0.01, ΔRMSEA ≤ 0.015, ΔSRMR ≤ 0.03) (54). These results indicate that the T1 and T2 second-order factors share the same measurement structure, supporting their comparability in the cross-lagged and latent moderation models. In this study, physical exercise behavior is measured using the product of three items as an observed indicator; as an observed variable, it does not involve the latent variable measurement model and therefore is not subject to longitudinal measurement invariance testing (55).

**Table 3:** Analysis of longitudinal measurement invariance.

Model	CFI	TLI	RMSEA	SRMR	ΔCFI	ΔRMSEA	ΔSRMR
Configural	0.987	0.982	0.046	0.029	–	–	–
Metric	0.984	0.981	0.048	0.049	0.003	0.002	0.020
Scalar	0.981	0.978	0.051	0.061	0.003	0.003	0.012
Strict	0.976	0.975	0.055	0.055	0.005	0.004	0.006

The model consisted of eight items loading on two first-order factors, which in turn loaded on one second-order factor. Configural invariance freely estimates all parameters; metric invariance constrains equal factor loadings across time; scalar invariance further constrains equal item intercepts; and strict invariance further constrains equal item residual variances.

### 3.4 Analysis of differences

Independent-samples t-tests were conducted for the variables, and the results are shown in Table 4: Subjective exercise experience (T1: *t* = 4.501, *p* < 0.001; T2: *t* = 6.676, *p* < 0.001), physical exercise behavior (T1: *t* = 6.553, *p* < 0.001; T2: *t* = 7.357, *p* < 0.001), and motivational beliefs (exercise self-efficacy: *t* = 5.528, *p* < 0.001; perceived value of exercise: *t* = 4.080, *p* < 0.001) were all significant. Male students’ average scores for subjective exercise experience, physical exercise behavior, and motivational beliefs were all higher than those of female students. In summary, significant gender differences in subjective exercise experience and physical exercise behavior were observed at both time points, with male students’ average scores higher than those of female students.

**Table 4:** Independent-samples *t*-tests for gender differences across variables.

Variable	Gender	*M* ± SD	Levene-test		*t*-test				
			*F*	*P*	*t*	*df*	*P*	95% CI	
								LLCI	ULCI
T1 Subjective exercise experience	Male	29.94 ± 6.253	1.120	0.291	4.501^2^	420.000	0.000	1.530	3.903
	Female	27.23 ± 6.121							
T2 Subjective exercise experience	Male	29.89 ± 5.774	0.177	0.674	6.676^2^	420.000	0.000	2.704	4.961
	Female	26.06 ± 6.012							
T1 Physical exercise behavior	Male	27.23 ± 21.247	29.757	0.000	6.553^1^	397.489	0.000	8.083	15.187
	Female	15.59 ± 14.961							
T2 Physical exercise behavior	Male	28.29 ± 20.440	27.247	0.000	7.357^1^	392.498	0.000	9.051	15.825
	Female	15.85 ± 13.976							
Exercise self-efficacy	Male	22.30 ± 5.547	0.023	0.879	5.528^2^	420.000	0.000	1.960	4.123
	Female	19.26 ± 5.751							
Perceived value of exercise	Male	25.13 ± 4.343	0.386	0.535	4.080^2^	420.000	0.000	0.897	2.565
	Female	23.40 ± 4.363							

^1^Heteroscedasticity (Levene’s test, *p* < 0.05); corrected *t*-values reported under the assumption of unequal variances; ^2^Homoscedasticity (Levene’s test, *p* > 0.05); *t*-values reported under the assumption of equal variances.

### 3.5 Cross-lagged analysis

A cross-lagged model of university students’ subjective exercise experience and physical exercise behavior was analyzed (Figure 3). In the model, physical exercise behavior was included as a manifest variable, while subjective exercise experience was included as a second-order latent factor. Results from the analysis of variance indicated significant gender differences for all variables; therefore, gender was included in the model as a control variable. The model fit was good: *χ^2^/df* = 1.5, CFI = 0.985, TLI = 0.981, RMSEA = 0.035, SRMR = 0.031. Subjective exercise experience at T1 significantly and positively predicted physical exercise behavior at T2 (*β* = 0.385, *p* < 0.001), and physical exercise behavior at T1 significantly and positively predicted subjective exercise experience at T2 (*β* = 0.336, *p* < 0.001). Cross-lagged path analysis indicated a bidirectional positive predictive relationship between physical exercise behavior and subjective exercise experience. Further Wald tests of the standardized effect sizes for the two cross-lagged paths revealed no statistically significant difference (Δ*χ^2^* = 0.284, *df* = 1, *p* = 0.594).

**Figure 3:**
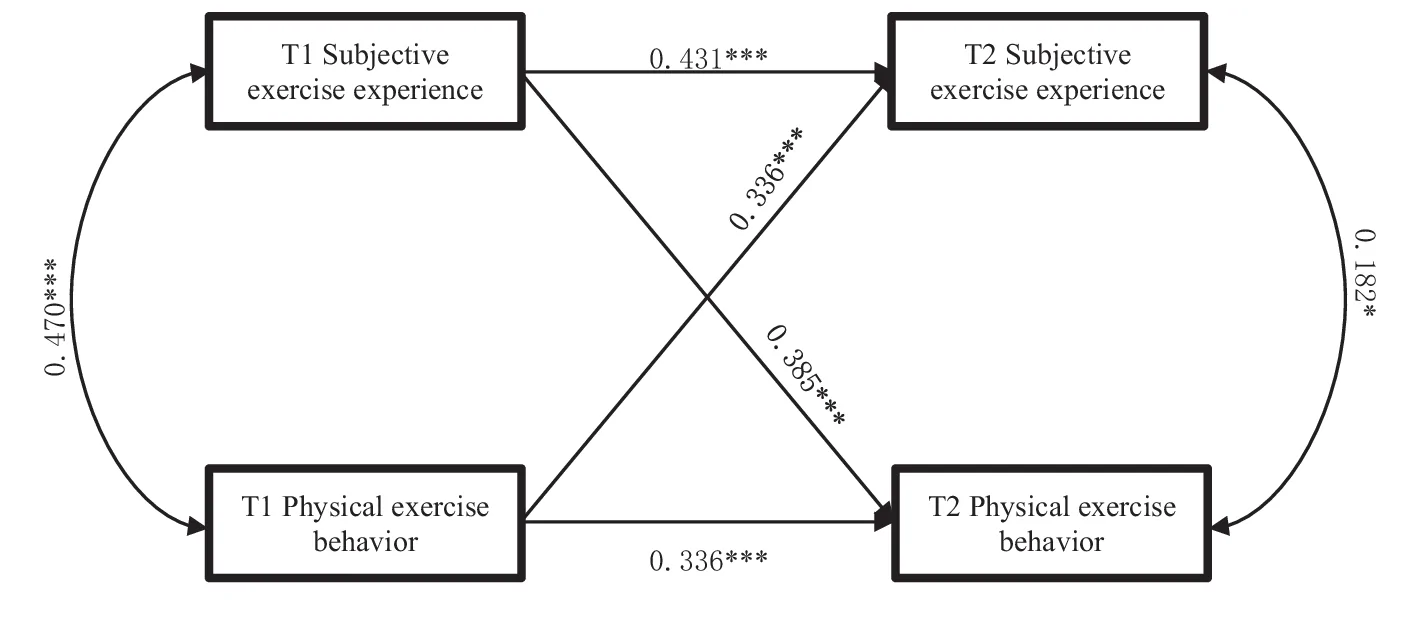
Cross-lagged model of subjective exercise experience and physical exercise behavior. *p < 0.05, **p < 0.01, ***p < 0.001.

### 3.6 The moderating role of motivational beliefs

To test the moderating effect of exercise self-efficacy, we included the interaction terms between exercise self-efficacy and T1 physical exercise behavior, as well as between exercise self-efficacy and T1 subjective exercise experience, in the cross-lagged model. To test the moderating effect of perceived value of exercise, the interaction terms between perceived value of exercise and T1 physical exercise behavior, and between perceived value of exercise and T1 subjective exercise experience, were, respectively, included in the cross-lagged model. Exercise self-efficacy and perceived value of exercise were both measured at T1 and treated as T1 moderators. In these models, the same second-order measurement model was used. The second-order latent factor representing overall subjective exercise experience was used to construct interaction terms with the T1 moderators. Interaction terms were constructed directly within the model using the XWITH command in Mplus. The model used robust maximum likelihood estimation (MLR), with TYPE = RANDOM, ALGORITHM = INTEGRATION, INTEGRATION = MONTECARLO, 2,000 integration points, two dimensions of numerical integration, and adaptive quadrature enabled. The manifest variables T1 physical exercise behavior and T2 physical exercise behavior were grand-mean centered, and latent variable means were fixed to 0, which is equivalent to centering. Interaction terms involving the latent variable subjective exercise experience were estimated via numerical integration within the LMS framework (56), and factor scores were not extracted to avoid two-step bias (57). No direct comparison of the relative strength of the two moderators was conducted because they were measured on different scales and were not tested within a single joint model. The results are shown in Figures 4, 5.

**Figure 4:**
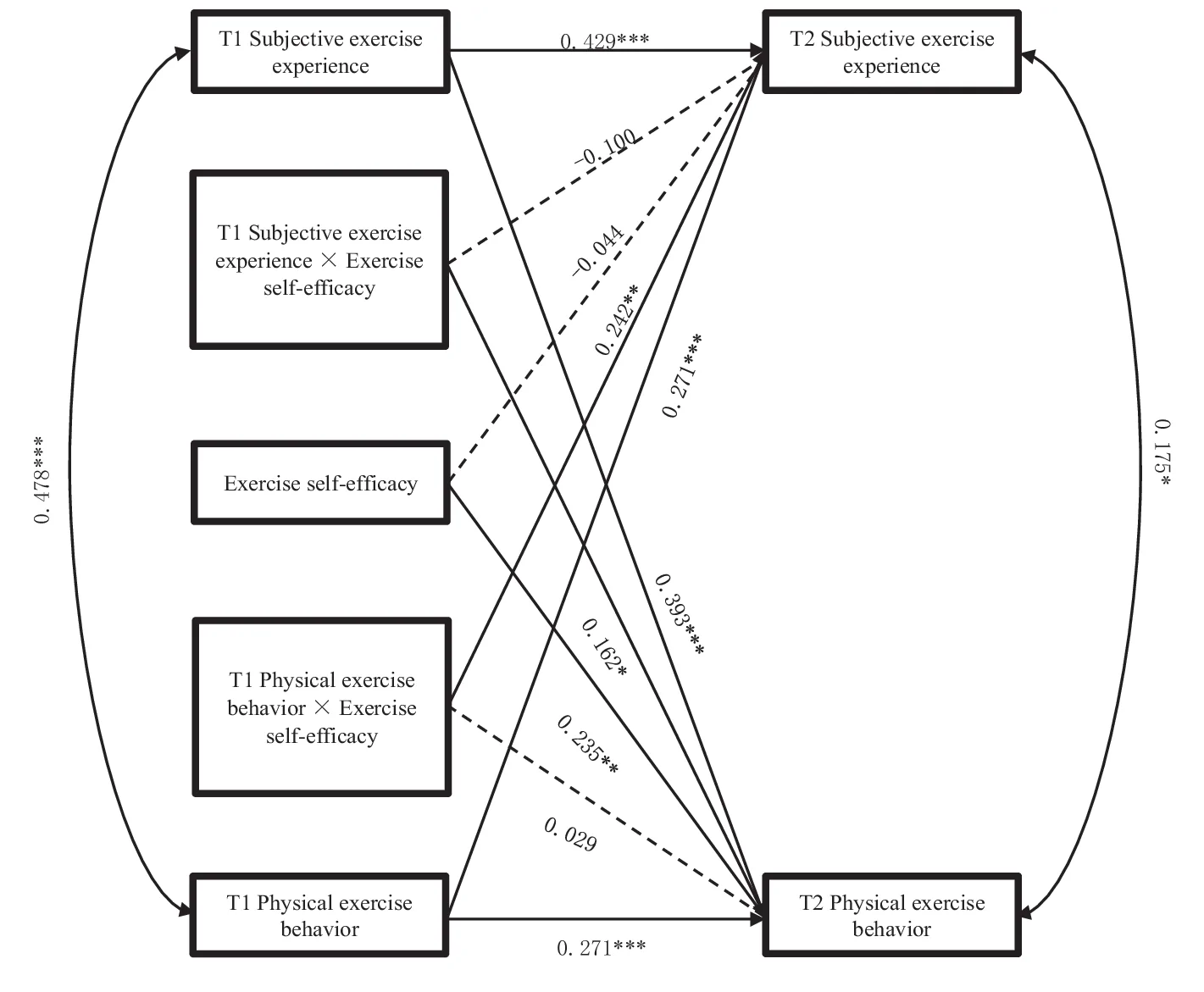
The moderating effect of exercise self-efficacy. *p < 0.05, **p < 0.01, ***p < 0.001; solid lines indicate significance, while dotted lines indicate non-significance.

**Figure 5:**
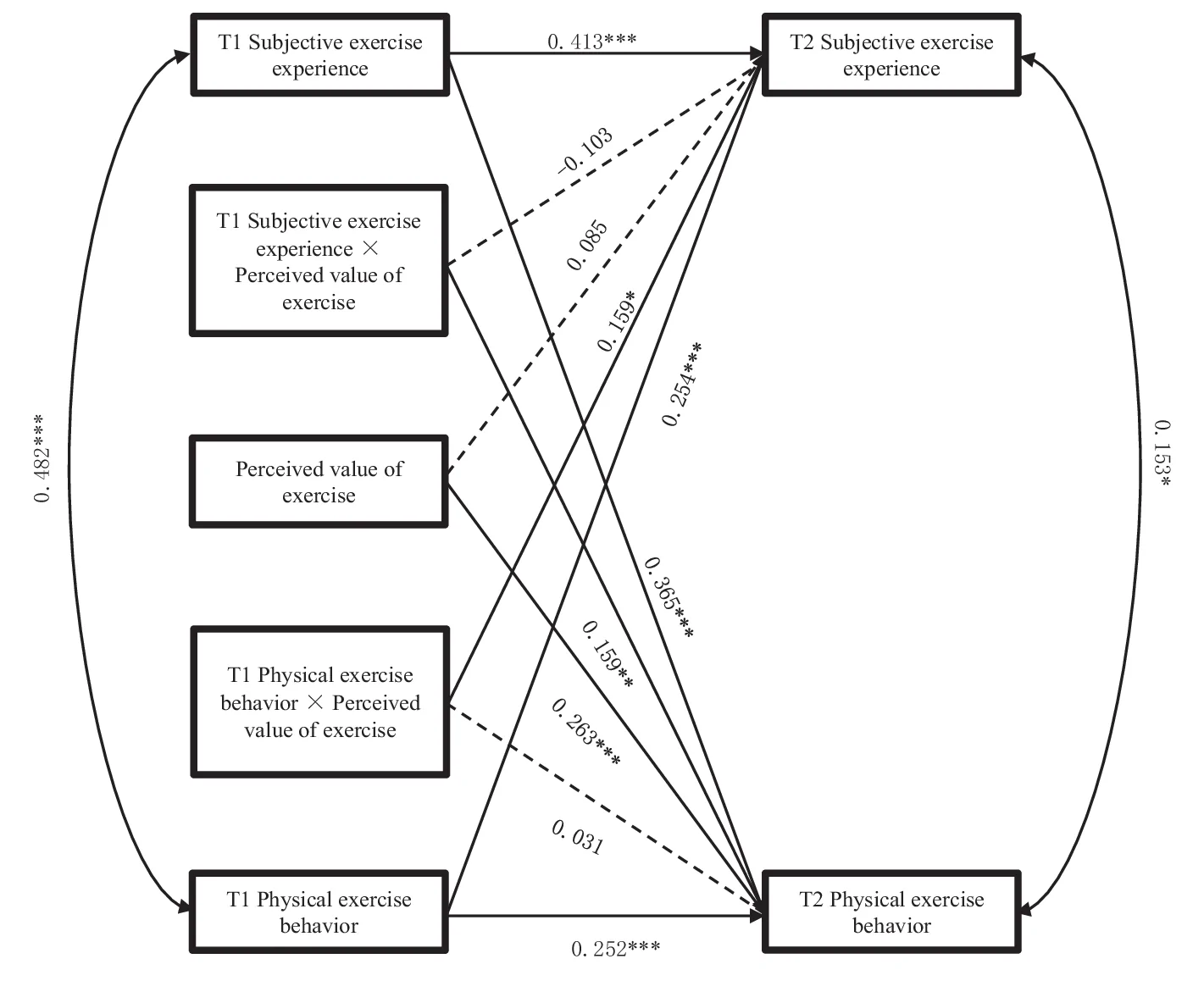
The moderating effect of perceived value of exercise. *p < 0.05, **p < 0.01, ***p < 0.001; solid lines indicate significance, while dotted lines indicate non-significance.

The results showed that the autoregressive path of university students’ subjective exercise experience was significant (*β* = 0.429, *p* < 0.001), and T1 subjective exercise experience still significantly predicted T2 physical exercise behavior (*β* = 0.393, *p* < 0.001). The interaction term between exercise self-efficacy and T1 subjective exercise experience had a significant positive predictive effect on T2 physical exercise behavior (*β* = 0.162, *p* = 0.021), but no significant predictive effect on T2 subjective exercise experience (*β* = −0.100, *p* = 0.333), indicating that exercise self-efficacy moderates the path “T1 subjective exercise experience → T2 physical exercise behavior.” Physical exercise behavior among university students still exhibited strong stability (*β* = 0.271, *p* < 0.001), and physical exercise behavior at T1 still significantly predicted subjective exercise experience at T2 (*β* = 0.271, *p* < 0.001). The interaction term between exercise self-efficacy and T1 physical exercise behavior had a significant positive predictive effect on T2 subjective exercise experience (*β* = 0.242, *p* = 0.002), but no significant predictive effect on T2 physical exercise behavior (*β* = 0.029, *p* = 0.643), indicating that exercise self-efficacy moderates the path from T1 physical exercise behavior to T2 subjective exercise experience.

The results showed that the autoregressive path of university students’ subjective exercise experience was significant (*β* = 0.413, *p* < 0.001), and that subjective exercise experience at T1 still significantly predicted physical exercise behavior at T2 (*β* = 0.365, *p* < 0.001); the interaction term between perceived value of exercise and T1 subjective exercise experience had a significant positive predictive effect on T2 physical exercise behavior (*β* = 0.159, *p* = 0.004), but no significant predictive effect on T2 subjective exercise experience (*β* = −0.103, *p* = 0.224), indicating that perceived value of exercise moderates the path ‘T1 subjective exercise experience → T2 physical exercise behavior’. Physical exercise behavior among university students continued to demonstrate strong stability (*β* = 0.252, *p* < 0.001), with T1 physical exercise behavior still significantly predicting T2 subjective exercise experience (*β* = 0.254, *p* < 0.001), the interaction term between perceived value of exercise and T1 physical exercise behavior had a significant positive predictive effect on T2 subjective exercise experience (*β* = 0.159, *p* = 0.035), but no significant predictive effect on T2 physical exercise behavior (*β* = 0.031, *p* = 0.695), indicating that perceived value of exercise moderates the path from T1 physical exercise behavior to T2 subjective exercise experience.

To clarify the direction of the moderating effects, a simple slope analysis was further conducted by categorizing the mean values of the moderator variables ±1 standard deviation into low, medium, and high levels; the results are shown in Table 5. For exercise self-efficacy, on the path from T1 subjective exercise experience to T2 physical exercise behavior, the conditional effects under low, medium, and high levels of self-efficacy were *b* = 5.775, 95% CI [1.821, 9.729], *p* = 0.004; *b* = 9.827, 95% CI [5.066, 14.588], *p* < 0.001; *b* = 13.879, 95% CI [6.280, 21.477], *p* < 0.001. On the path from T1 physical exercise behavior to T2 subjective exercise experience, the conditional effects under low, moderate, and high self-efficacy were *b* = 0.001, 95% CI [−0.006, 0.008], *p* = 0.785; *b* = 0.009, 95% CI [0.005, 0.014], *p* < 0.001; *b* = 0.018, 95% CI [0.011, 0.025], *p* < 0.001. Regarding the perception of the value of exercise, in the path from subjective exercise experience at T1 to physical exercise behavior at T2, the conditional effects under low, medium, and high value perceptions were as follows: *b* = 5.177, 95% CI [1.551, 8.802], *p* = 0.005; *b* = 9.173, 95% CI [4.365, 13.981], *p* < 0.001; *b* = 13.169, 95% CI [6.004, 20.335], *p* < 0.001. The values showed a numerically increasing trend, and the significant interaction term indicated that the moderating effect was statistically significant. On the path from T1 physical exercise behavior to T2 subjective exercise experience, the conditional effects under low, medium, and high perceived value were *b* = 0.003, 95% CI [−0.005, 0.011], *p* = 0.437; *b* = 0.009, 95% CI [0.004, 0.013], *p* < 0.001; *b* = 0.014, 95% CI [0.009, 0.019], *p* < 0.001. Among these, the conditional effects of the “behavior → experience” path under both low self-efficacy and low perceived value were not statistically significant. However, the significant interaction terms indicated that as the level of the moderators increased, the conditional effects of both cross-lagged paths generally showed a significant increasing trend. Specifically, the positive effect of physical exercise behavior on subjective exercise experience became stronger and significant at medium and high levels of self-efficacy and perceived value.

**Table 5:** Results of simple slope analysis for moderation effects.

Moderator variable	Path	Level	*b*	*SE*	*t*	*p*	95% CI
Exercise self-efficacy	T1 Experience → T2 Behavior	Low (−1SD)	5.775	2.017	2.863	0.004	[1.821, 9.729]
		Medium (Mean)	9.827	2.429	4.046	<0.001	[5.066, 14.588]
		High (+1SD)	13.879	3.877	3.58	<0.001	[6.280, 21.477]
	T1 Behavior → T2 Experience	Low (−1SD)	0.001	0.004	0.273	0.785	[−0.006, 0.008]
		Medium (Mean)	0.009	0.002	4.253	<0.001	[0.005, 0.014]
		High (+1SD)	0.018	0.004	4.917	<0 0.001	[0.011, 0.025]
Perceived value of exercise	T1 Experience → T2 Behavior	Low (−1SD)	5.177	1.850	2.799	0.005	[1.551, 8.802]
		Medium (Mean)	9.173	2.453	3.74	<0 0.001	[4.365, 13.981]
		High (+1SD)	13.169	3.656	3.602	<0 0.001	[6.004, 20.335]
	T1 Behavior → T2 Experience	Low (−1SD)	0.003	0.004	0.777	0.437	[−0.005, 0.011]
		Medium (Mean)	0.009	0.002	3.713	<0.001	[0.004, 0.013]
		High (+1SD)	0.014	0.002	5.614	<0.001	[0.009, 0.019]

b represents the unstandardized simple slope; SE represents the standard error; t = b/SE; 95% CI represents the confidence interval.

## 4 Discussion

### 4.1 Analysis of the characteristics

The study tested for differences in subjective exercise experiences and physical exercise behavior across two stages using gender as a grouping variable. The results revealed gender differences in both subjective exercise experiences and physical exercise behavior across the two stages, with male students scoring higher on average than female students. These findings are consistent with those reported by Dong et al. (22). According to social role theory, social expectations associated with traditional gender roles constitute deep-seated cultural motivators; at the sociocultural level, there is a widespread attitude of encouragement and affirmation toward men’s participation in sports (58); athletic ability is often viewed as an important component of male attractiveness, whereas role expectations for women tend to emphasize quietness and reserve, which to some extent weakens female students’ intrinsic motivation to participate in physical exercise (59). This not only results in their overall exercise frequency and intensity being lower than that of male students but also subtly influences female students’ perceptions and evaluations of the exercise process, reducing the likelihood of positive experiences (10).

### 4.2 Cross-lagged analysis

Correlation analysis revealed that university students’ subjective exercise experiences and physical exercise behavior at both time points were significantly positively correlated, demonstrating both concurrent correlation at the same time point and longitudinal correlation across time points. This result supports research hypothesis H1 and is consistent with previous studies (23). A cross-lagged model controlling for gender showed that subjective exercise experience at T1 significantly and positively predicted physical exercise behavior at T2, and physical exercise behavior at T1 also significantly and positively predicted subjective exercise experience at T2. These results are consistent with the conclusions of previous studies (21). This indicates that there is a significant bidirectional positive predictive relationship between university students’ subjective exercise experience and physical exercise behavior; at the observational level, the two exhibit a bidirectional prospective association and may be mutually conditioning. Research hypotheses H2 and H3 were both supported.

From the perspective of Self-Determination Theory, when university students experience abundant energy, positive emotions, and low stress during exercise, this experience is internalized as an autonomous motivation. Consequently, subsequent exercise is no longer driven by external pressure but stems from the individual’s anticipation and pursuit of positive experiences, thereby increasing the likelihood of engaging in exercise (60). From the perspective of emotional feedback theory, when individuals decide whether to engage in future exercise, they mentally simulate and anticipate the emotional outcomes that the behavior may bring (61). Past positive subjective exercise experiences form the informational basis for optimistic emotional expectations, making individuals more inclined to believe that the next exercise session will also bring pleasure and satisfaction; this anticipatory process, in turn, promotes action initiation by influencing behavioral intentions (62, 63). At the same time, relevant scholars argue that regular and sustained exercise behavior not only improves an individual’s emotional state through physiological pathways—such as enhanced cardiopulmonary function and neuroendocrine regulation—but also strengthens an individual’s physical self-esteem and sense of control through psychosocial channels, such as improved physical fitness and mastery of skills (64). This, in turn, optimizes subjective experiences during exercise and elevates the level of positive experiences (65).

In light of this, physical exercise promotion and intervention programs targeting university students should adopt a two-pronged approach, focusing both on fostering positive experiences and on cultivating consistent exercise habits. For example, in physical education classes or fitness instruction, exercise routines that are engaging, moderately challenging, and provide immediate positive feedback can help students gain pleasurable subjective experiences early on, thereby encouraging subsequent independent exercise. At the same time, positive subjective exercise experiences should be cultivated by enhancing the enjoyment and sense of accomplishment during the exercise process, thereby achieving the goal of experience-driven behavioral persistence. For example, strategies such as behavioral contracts and the cultivation of micro-habits can encourage students to first engage in regular exercise and gradually improve their subjective experiences. Future intervention studies can examine the effectiveness of strategies that balance experience optimization with behavioral accumulation. Only if a mutually reinforcing relationship between experience and behavior is observed in experiments can the “positive feedback loop” hypothesis be supported.

### 4.3 The moderating role of motivational beliefs

When motivational beliefs were included as moderator variables in the cross-lagged model, the results showed that neither type of motivational belief exhibited a moderating effect in the autoregressive paths (T1 subjective exercise experience → T2 subjective exercise experience, T1 physical exercise behavior → T2 physical exercise behavior); thus, Hypotheses H4a, H4b, H5a, and H5b were not supported. Both types of motivational beliefs exhibited significant positive moderating effects in the cross-lagged paths (T1 subjective exercise experience → T2 physical exercise behavior; T1 physical exercise behavior → T2 subjective exercise experience), supporting Hypotheses H6a, H6b, H7a, and H7b. This indicates that motivational beliefs are not the primary factors altering the stability of individuals’ existing experiences and behaviors, but rather play a key moderating role in the process of mutual transformation between experience and behavior.

Motivational beliefs did not show a significant moderating effect on the autoregressive paths. Specifically, regardless of whether university students held high or low exercise self-efficacy and perceived value of exercise, the cross-temporal autoregressive paths of their subjective exercise experience and physical exercise behavior were not significantly moderated. However, it should be noted that this result only indicates that no significant moderation was detected in the two-wave data of this study; it cannot be concluded that motivational beliefs have no influence on the continuity of subjective exercise experience or the inertia of physical exercise behavior. This non-significant result may also be related to the relatively long interval between the two waves, sample size, measurement methods, or a small effect size, and future research with larger samples or more waves is needed to further examine this issue. One possible but untested explanation is that when behaviors or affective responses become automated through repetition to a certain degree, their maintenance may rely more on situational cues and procedural memory than on conscious motivational evaluation processes (36). Related research suggests that as habit strength increases, the predictive power of conscious intentions for behavior may weaken, and behavioral execution may gradually shift to automatic pathways. In individuals who have formed an exercise habit, the predictive power of self-efficacy for behavior may also be greatly diminished (37). However, habit strength was not measured in this study; therefore, the above explanation is only speculative and cannot be regarded as a confirmed mechanism of the present findings. Regardless of whether university students believe they are capable of exercising or endorse the value of exercise, established patterns of experience and behavioral inertia may persist in a relatively autonomous manner; however, whether strengthening motivational beliefs is sufficient to further amplify their autoregressive effects still needs to be verified in future research. Motivational beliefs exhibit a significant positive moderating effect on the cross-lagged path. Specifically, the cross-lagged predictive relationship between subjective exercise experience and physical exercise behavior significantly depends on an individual’s motivational beliefs. This finding aligns with the core tenet of Bandura’s social cognitive theory—the triadic interaction determinism—which posits that there is a dynamic interaction among individual factors, behavior, and the environment, with all three being causally interrelated. Motivational beliefs may serve as a conditional moderator of the prospective association between subjective exercise experience and physical exercise behavior (31). Positive experiences may not automatically translate into sustained participation. This transformation may occur more readily when individuals simultaneously value the benefits of exercise. This mechanism requires further experimental testing (27, 66). Actual exercise behavior does not necessarily lead to stable intrinsic pleasure; only when individuals experience a sense of competence during the activity can the positive feelings derived from exercise be truly internalized (20).

In light of this, practical physical exercise interventions for university students should not treat exercise self-efficacy and the perception of exercise value as separate constructs but should instead focus on enhancing both simultaneously. Specifically, by setting progressive challenge tasks, providing role models, and offering encouraging feedback, educators can boost students’ confidence in overcoming obstacles such as time constraints, physical limitations, and environmental barriers, thereby enhancing their exercise self-efficacy. At the same time, through health education, classroom discussions, and goal-setting, educators can help students understand the multifaceted benefits of physical exercise—including physical health, emotional regulation, academic development, and social interaction—thereby deepening their perception of the value of exercise. Future intervention studies could attempt to simultaneously enhance exercise self-efficacy and perceived value, and test whether this can strengthen the positive association between experience and behavior.

## 5 Research shortcomings and future prospects

Although this study employed a two-phase cross-lagged design and preliminarily revealed the longitudinal relationship between university students’ subjective exercise experience and physical exercise behavior, as well as the moderating role of motivational beliefs, the following limitations remain: (1) This study employed convenience sampling, selecting only first-year undergraduate students (non-physical education majors) from 18 classes at 6 universities in Nanchang and Jiujiang, Jiangxi Province. The findings may be influenced by regional cultural factors and the characteristics of the college adjustment period, making it difficult to directly generalize the results to other populations. Future research should adopt probability sampling to cover different types of institutions, grade levels, and majors in order to enhance the external validity of the conclusions. (2) This study relied primarily on self-report measures. Although the reliability and validity of the scales were good, self-reports may still be subject to recall bias and common-method bias. Future research could incorporate objective behavioral indicators, such as accelerometers and heart rate monitors, to reduce recall bias and further examine the impact of common-method bias on the relationships among variables. (3) In this study, students were nested within classes and schools. Although the ICC(1) and design effects were small, the study may still have underestimated the effects—such as standard errors—arising from the shared environmental factors at the class and school levels. Future research should employ multilevel structural equation modeling to incorporate random effects at the class and school levels into the analysis. (4) Although the two-wave cross-lagged model can test predictive relationships across time, it is difficult to distinguish between stable differences between individuals and genuine changes within individuals. Future studies should employ intensive longitudinal tracking across three or more waves, utilizing RI-CLPM or alternative cross-lagged models, and incorporating more time-varying covariates. (5) The Monte Carlo power assessment for the main cross-lagged paths in this study was post-hoc and data-dependent, because the population parameters were derived from the observed sample. Therefore, it cannot serve as an independent justification of sample-size adequacy; it only indicates that, conditional on the observed effect sizes, the main cross-lagged paths could be estimated with high power. No formal power simulation was conducted for the latent interaction/moderation effects. Future research should conduct power analyses specifically for latent interaction models to further validate the moderation findings of this study. In summary, future research should further improve in areas such as sample representativeness, objectivity, multilevel modeling, intensive longitudinal design, control of residual confounders, and intervention validation, in order to more robustly reveal the dynamic relationship between subjective exercise experiences and physical exercise behavior, and to provide evidence for targeted physical activity interventions for different populations.

## 6 Conclusion

This study employed a two-wave cross-lagged design to examine the longitudinal relationship between subjective exercise experience and physical exercise behavior among university students, as well as the moderating role of motivational beliefs. The results indicate that gender differences exist in both subjective exercise experience and physical exercise behavior among university students; there is a longitudinal relationship across time between subjective exercise experience and physical exercise behavior; and motivational beliefs exert a significant moderating effect on the prospective predictive pathways between subjective exercise experience and physical exercise behavior.

## Data Availability

The raw data supporting the conclusions of this article will be made available by the authors, without undue reservation.
